# Supplementary material for: PopPK and PBPK Models Guide Meropenem Dosing in Critically Ill Children with Augmented Renal Clearance
Source: Pharmaceutics. 2025 Nov 29;17(12):1544. doi: 10.3390/pharmaceutics17121544 (PMC12736838; doi:10.3390/pharmaceutics17121544)
Supplement: Supplementary file 1 [file pharmaceutics-17-01544-s001.zip › pharmaceutics-3966842-supplementary.pdf]

Supplementary Material

Table S1. Subject demographics of published adult data used for physiologically based pharmacokinetic (PBPK) model development and evaluation

Number of Subjects	Age (Years)[a]	Average Bodyweight (kg)[a]	Average Height (cm)[a]	Dose (g)	Infusion duration (min)	Available Data Format	Used for	Reference (PMID)
12	26<br>(19–45)	74<br>(68–87)	179<br>(170–184)	0.5	30	Mean ± SE	ME[b]	2808215
5	28.8<br>(18–39)	82.1<br>(72.9–87.8)	177<br>(165–185)	0.5	30	Mean ± SE	ME	8543497
8	20–34	68.6 ± 7.7	-	0.5	30	Mean ± SD	MD[c]	1510440
10	26.8<br>(21–42)	86.2<br>(68–118)	180<br>(168–188)	0.5[c]	30	Mean ± SD	ME	12709358

[a] mean (min, max), [b] MD model development, [c] ME model evaluation.

Table S2. Characteristics of enrolled patients with severe pneumonia

GFR (mL/min) Classification	Age (years)	Weight (kg)	Duration (h)	Infusion Interval(h)	Dose (mg)
>120	41-74	56-108	3	8	1000
	15-67	51-128	0.5		
90-120	52-73	68-83	3		
60-89	57-84	57-74	3		
	58-73	75-90	0.5		
30-59	51-84	49-90	3		
	69-88	52-99	0.5		
<30	71-82	72-84	3		
	62-81	74-79.5	0.5		

Table S3. Physicochemical and Biopharmaceutical Properties of Meropenem Used in the PBPK Models.

Parameters	Reported Values	Final Values
**Physicochemical parameters**		
**Molecular weight**	383.5g/mol	383.5g/mol
**Solubility**	5.63mg/mL [41]	5.63mg/mL
**pKa**	3.47 [42]	3.47
	7.4 [32]	7.4
**Lipophilicity(logP)**	-1.39 [11]	-1.39
**Partition coefficients**	Rodgers and Rowland[43]	Rodgers and Rowland
**Cellular permeabilities**	-	PK-Sim® Standard
**Pharmacokinetic properties**		
**Biliary clearance**	6.5E-04L/h/kg[11]	6.5E-04L/h/kg
**OAT3 Km**	850µM [11]	850µM
**OAT3 V_{max}**	600µmol/L/min [11]	82.48µmol/L/min[a]

**Hypothetical renal efflux transport protein Vmax**	200µmol/L/min [11]	21.92µmol/L/min[a]
**Hypothetical renal efflux transport protein Km**	1500µM [11]	1500µM
**DPEP 1 Clearance**	First-order intrinsic clearance,0.02L/min [11]	0.037L/min[a]
**f_u**	0.98 [41]	0.98
**Protein binding partner**	Albumin	Albumin

DPEP1 dehydropeptidase 1 or renal dipeptidase 1, **f_u** unbound fraction, Km Michaelis–Menten constant, OAT3 organic anion transporter subfamily 3, pKa acid dissociation constant, Vmax maximum velocity of transport/reaction

a Optimized values

Table S4. Simulated and Observed Pharmacokinetic Parameters for Meropenem After Intravenous in Healthy Adults

Reference (PMID)	Age (range)	Observed mean		Simulated mean (95%)		Ratio	
		AUC_{t_end}(mg·h/L)	C_{max}(mg/L)	AUC_{t_end}(mg·h/L)	C_{max}(mg/L)	AUC_{t_end}	C_{max}
**8543497**	**28.8 (18-39)**	26.02	23.38			0.79	0.97
**1510440**	**20-34**	37.43	34.84	32.89 (24.6-42.27)	24.97 (20.98-28.5)	1.14	0.97
**12709358**	**26.8 (21-42)**	23.81	24.26			0.72	0.97
**2808215**	**26 (19-45)**	29.85	24.77	30.85	28.7	0.97	0.86

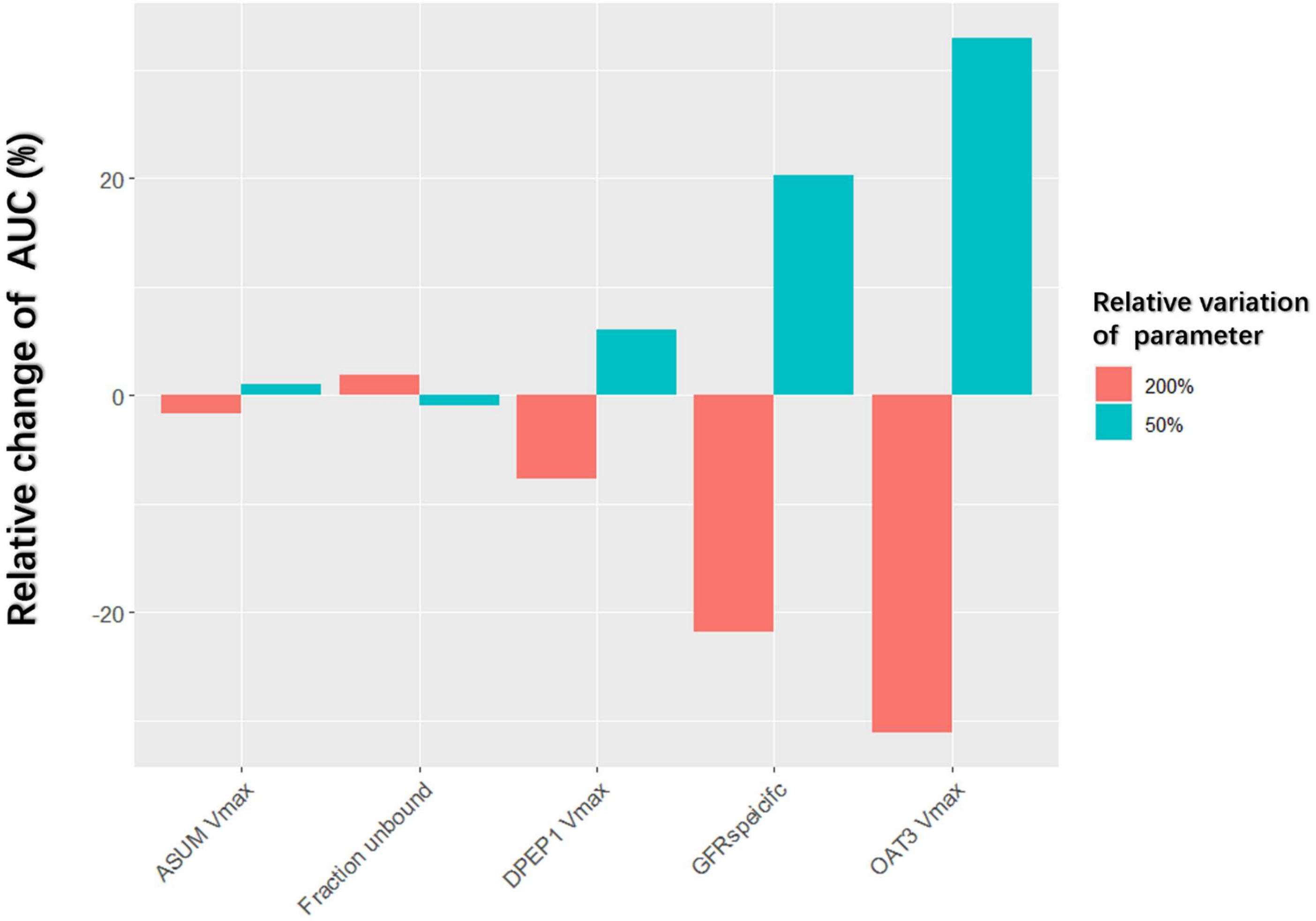

Figure S1. Local Sensitive Analyze. Each parameter was systematically varied within range of 50% to 200% of the baseline value.

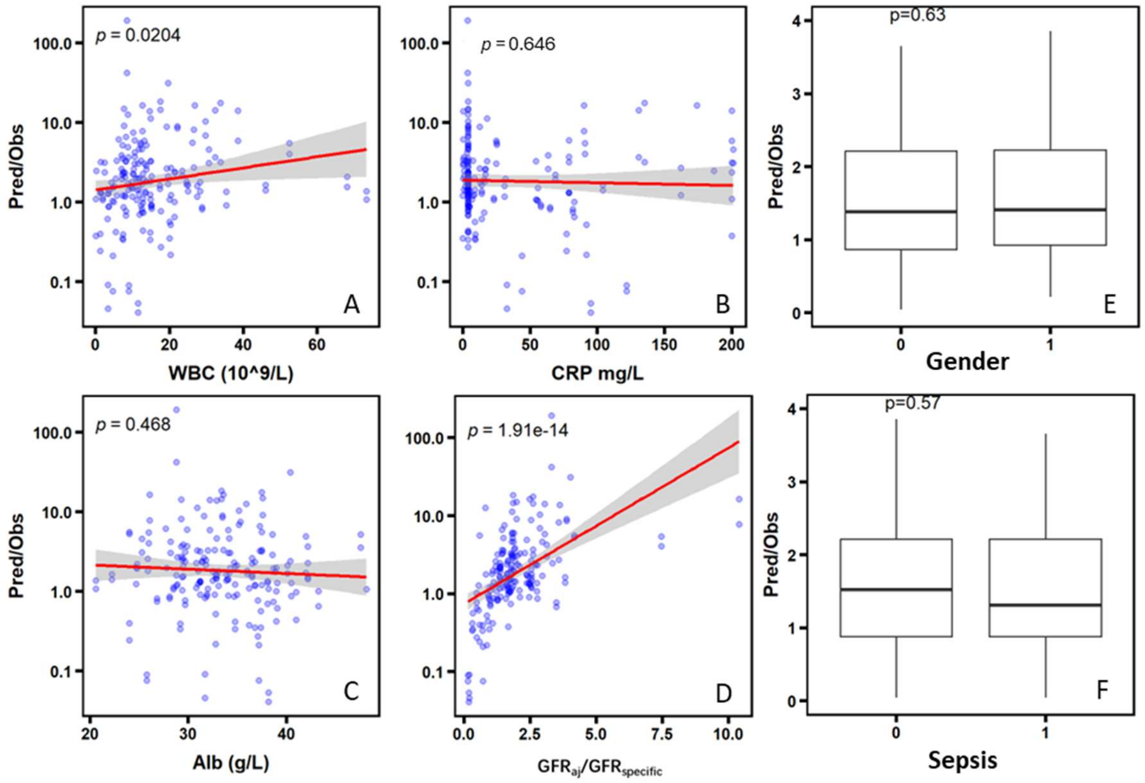

Figure S2. The correlation of model predictive accuracy with demographic characteristic of patients.

Pearson correlation analysis was used for continuous variables and logistic regression was used for binary variables in correlation analysis. A Pred/Obs vs WBC, B Pred/Obs vs Alb, C Pred/Obs vs CRP, D Pred/Obs vs ratio of GFR_{aj} and $GFR_{specific}$, E The relationship between gender and Pred/Obs(0 Male, 1 Female) , F The relationship between gender and sepsis(0 diagnosed without sepsis, 1 diagnosed with sepsis). Pred/Obs: Ratio of predicted concentration and observed concentration; WBC: white blood count; CRP: C-reactive protein. Pearson's correlation analysis was used for continuous variables, and logistic regression was used for binary variables in correlation analysis.

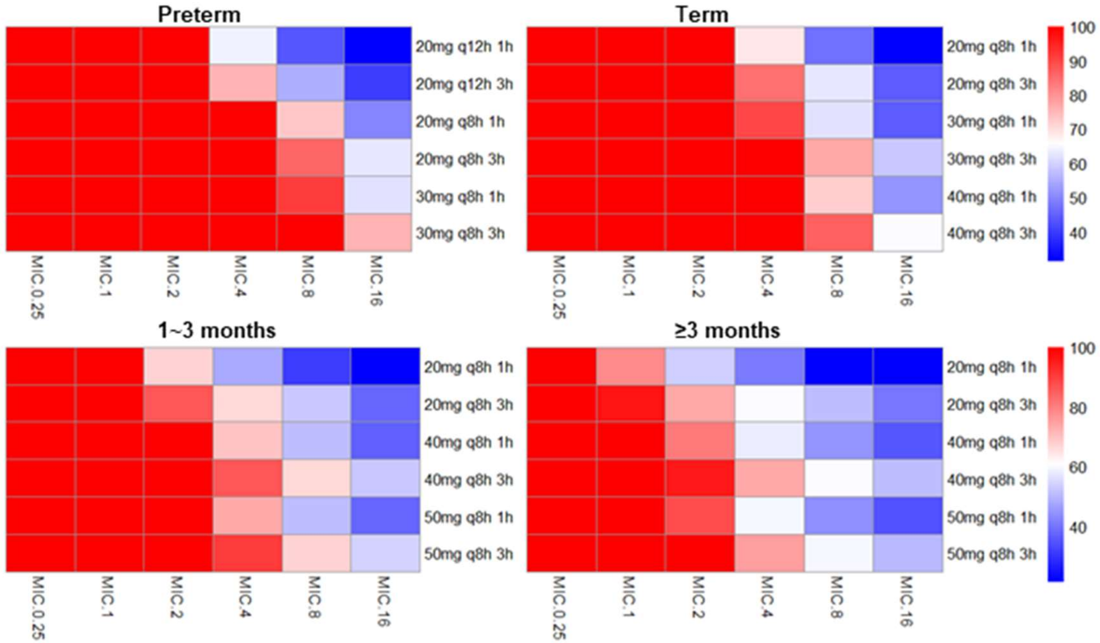

Figure S3. Target attainment analysis performed using the final physiologically based pharmacokinetic model to evaluate the different dose regimens.

500 virtual pediatric patients for each age cohort were dosed with six intravenous regimens. The percentage of the dosing interval for which the meropenem steady-state unbound plasma concentration was above the minimum inhibitory concentrations (MIC) of 0.25–16 mg/L was calculated for 90% of virtual pediatric patients and plotted against MIC on the x axis.
